# What can we learn about bone density in COPD patients from a chest CT? A systematic review

**DOI:** 10.3325/cmj.2024.65.440

**Published:** 2024-10

**Authors:** Danica Vuković, Danijela Budimir Mršić, Kristian Jerković, Tade Tadić

**Affiliations:** 1 Clinical Department of Diagnostic and Interventional Radiology, University Hospital Split, Split, Croatia; 2 School of Medicine, University of Split, Split, Croatia; 3 University Department of Health Studies, University of Split, Split, Croatia

## Abstract

We systematically reviewed the current research literature to 1) investigate whether there was a difference in bone mineral density (BMD) between chronic obstructive pulmonary disease (COPD) patients and non-COPD controls, 2) determine the influence of severity and subtype of COPD on BMD, and 3) determine the risk factors for lower BMD in COPD patients. The Web of Science and PubMed databases were searched on September 25, 2023. Studies where BMD was evaluated with computed tomography (CT) or quantitative CT in patients with COPD were included in the review. We collected data on the number of COPD patients, the average age, average body mass index, average predicted forced expiratory volume in one second (%) or Global Initiative for Chronic Obstructive Lung Disease stage, the average of low attenuation areas, the use of corticosteroid therapy, the use of osteoporosis therapy, the average BMD, and the location of BMD measurement. Twelve studies met our review criteria. Although in several studies COPD was associated with a decreased BMD, most of the studies suggested that COPD, especially in its milder forms, was not strongly associated with osteopenia or osteoporosis of the thoracic and lumbar spine.

Chronic obstructive pulmonary disease (COPD) is a lung disease characterized by an accelerating and irreversible airflow limitation. A hallmark of the disease is an abnormal and overly excessive inflammatory response of the lung tissue to noxious particles and gases (1). The Global Initiative for Chronic Obstructive Pulmonary Disease (GOLD) groups patients with COPD into four categories based on symptoms and exacerbation history (A to D), and into GOLD stage 1 to 4 based on forced expiratory volume in one second (FEV_1_) (2). Although COPD is predominantly a pulmonary illness, it leads to important extrapulmonary complications including loss of arterial compliance (progressive arterial stiffness), skeletal muscle wasting with consequential atrophy, systemic hypertension, and osteoporosis (1,2).

A direct link between COPD and osteoporosis is not yet clearly established, but it may include effects of cigarette smoke on bone tissue, decreased or lack of physical activity, poor nutritional profile, glucocorticoid use, and chronic inflammation (3). Osteoporosis is characterized by decreased bone strength as a result of declining micro-architectural integrity and low bone mineral density (BMD) (4). Individuals with osteoporosis are more likely to experience bone fracture (radial, hip, vertebral body), which significantly increases their morbidity and mortality. This is especially important for COPD patients, since vertebral fractures can worsen their already compromised lung function. The primary predictor of BMD loss is age, but sex, obesity, and a host of other disorders also play a role (4-6).

BMD is measured with several noninvasive modalities. The gold standard is dual-energy x-ray absorptiometry (DXA), but in recent decades BMD has been opportunistically estimated with quantitative CT (QCT) scanning (expressed in mg/mL) and quantitative ultrasound. It can also be measured with standard CT (expressed in Hounsfield units), which excellently correlates with the reference DXA measurements (7). CT is commonly used in the examination of pulmonary diseases, including COPD, to determine the nature and extent of the condition (8). CT scans not only provide information about the lung parenchyma, but also on atherosclerosis, BMD, and the amount of visceral fat (9). With this in mind, the assumption is that CT may serve as a surrogate method for BMD measurement instead of DXA, since it may provide more valuable information within a single examination, thus reducing the radiation exposure. Romme et al showed that BMD measured on chest CT was strongly associated with BMD measurements on DXA in a COPD population (7). However, in the literature, we have not identified any systematic review on BMD in patients with COPD evaluated by CT or QCT.

Therefore, the aim of this systematic review was to summarize current research on BMD in patients with COPD evaluated by CT or QCT. Specifically, we investigated 1) if there is a difference in BMD values between COPD patients and non-COPD controls, 2) the influence of severity and subtype of COPD on BMD, and 3) risk factors for lower BMD in COPD patients.

## MATERIALS AND METHODS

### Search strategy

The systematic review was conducted according to the 2020 Preferred Reporting Items for Systematic Reviews and Meta-analyses (PRISMA) checklist. Web of Science (WOS) and Medline/PubMed databases were searched on September 25, 2023. A combination of the following search words was used: “emphysema,” “COPD,” “bone mineral density,” and “CT.” The inclusion criteria were as follows: original research articles, English-language articles, studies involving living human subjects, bone density measurement performed with standard CT in HU or QCT in mg/mL.

**Figure 1:**
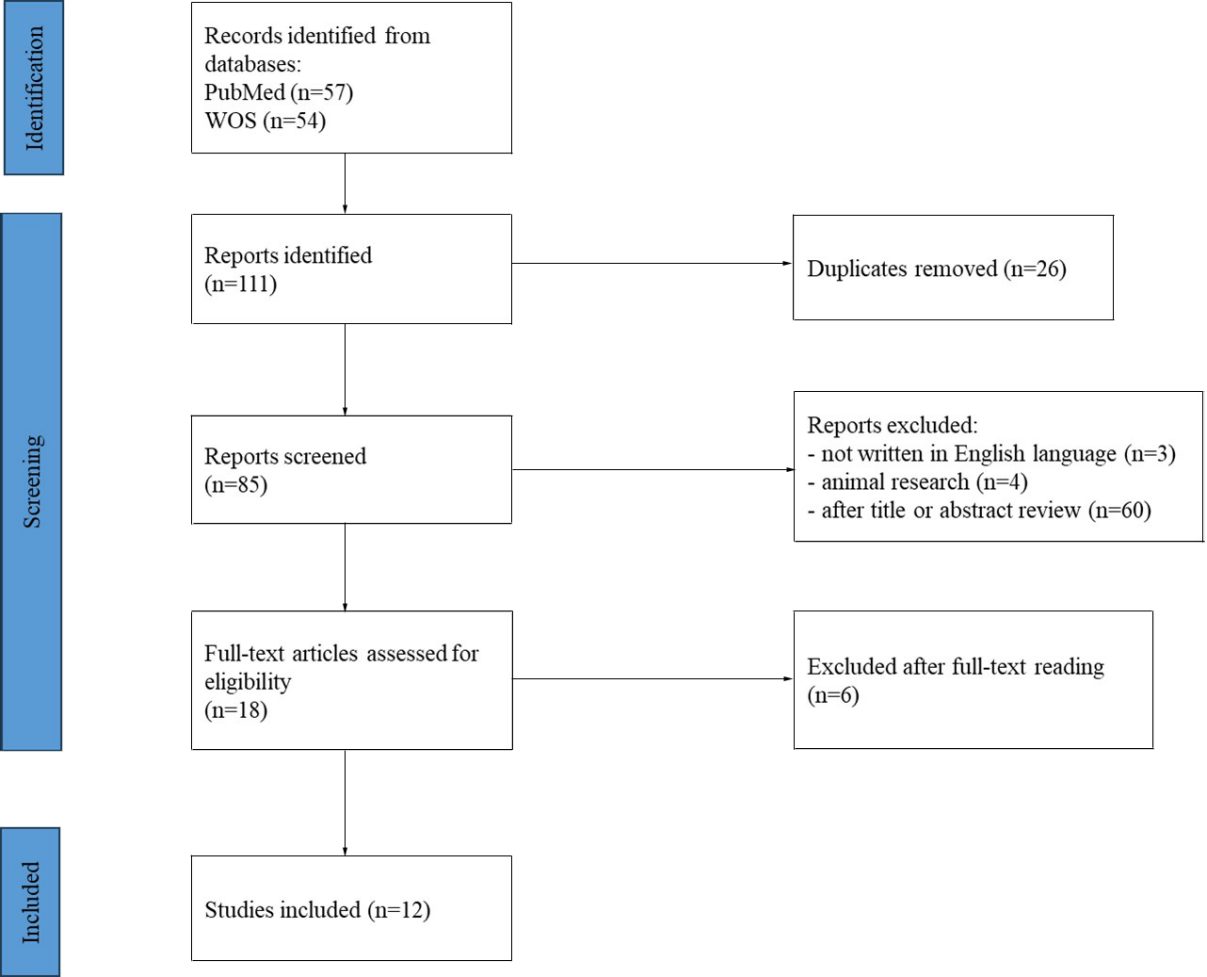
Study flowchart.

The initial search (performed by DV) resulted in 111 published papers: 57 from Medline and 54 from WOS. Twenty-six duplicates were removed. Seven studies were excluded as they were performed on animals or not written in English. After abstract and title screening (DV and DBM), further 60 studies were excluded. Both authors read the full text of the remaining studies and excluded 6 studies. Finally, 12 studies were included in the systematic review (10-21). The study selection strategy is presented in Figure 1.

### Data analysis

After a detailed analysis of the 12 selected studies, the following data were extracted: the name of the first author, the year of publication, characteristics of study participants, study design, exclusion criteria, research question, the number of COPD patients, the average age, average BMI, average FEV1% predicted or GOLD stage, average of low attenuation areas (LAA%), the use of corticosteroid therapy, the use of osteoporosis therapy, the average BMD, the location of BMD measurement, and fractured vertebrae excluded from measurement. Given the excessive heterogeneity of the study objectives and study designs, statistical analysis was not performed.

## RESULTS

### Studies on the difference in BMD between COPD and non-COPD patients

Out of 12 studies included, five investigated the difference in BMD between COPD and non-COPD patients (Table 1). Four of these studies measured BMD with standard CT (expressed in HU), while one study measured BMD with QCT (expressed in mg/mL). Three studies showed a lower BMD in the COPD group than in the non-COPD group. De Jong et al (3) found lower BMD in patients with COPD than in controls (100.7 HU vs 108.9 HU, *P* < 0.001), while Wilson et al (10) found lower BMD in COPD patients (120.1 ± 44.4 mg/cm^3^) than in smoking (148.9 ± 47.8 mg/cm^3^) and non-smoking controls (137.8 ± 39.6 mg/cm^3^, *P* < 0.001) (10). Romme et al found similar findings (patients: 164.9 ± 49.5 HU; smoking non-COPD controls: 183.8 ± 46.1 HU; non-smoking non-COPD controls: 212.1 ± 54.4 HU, *P* < 0.001), but after adjustment for age, sex, and pack-years of smoking, the difference was not significant (*P* = 0.206) (11). Conversely, Pompe et al (12) and van Dort et al (13) found no difference between the groups. However, van Dort et al found a significant difference in BMD between COPD and non-COPD participants only in men at the twelfth thoracic vertebra (T12) (*P* = 0.03) (13).

**Table 1:** Difference in bone density between chronic obstructive pulmonary disease (COPD) and non-COPD patients*

First author and year	Study design	Participants	Exclusion criteria	Research question	Location of BMD measurement and method	Fractured vertebra excluded	Outcomes regarding BMD
De Jong et al (3), 2014	cross-sectional	NELSON trial n = 1140	self-reported moderate or poor health; recent CT scan of the thorax; current or previous cancer history; weight more than 140 kg	prevalence of vertebral fractures; association of BMD and vertebral fractures with COPD status and smoking	L1 vertebra and, if fractured, the closest one in HU; ROI of only homogenous trabecular bone	yes	BMD was lower in COPD patients (100.7 HU vs 108.9 HU, *P* < 0.001) vertebral fracture prevalence was similar in COPD patients and non-COPD patients (9.6% vs 8.3%, *P* = 0.430)
Wilson et al (10), 2022	cohort study	COPDGene n = 9703	N/A	impact of BMD on clinical and survival outcomes in COPD patients	mean value of estimated bone attenuations of the vertebra T6-L1 (in mg/cm^3^)	yes	COPD patients (GOLD 1-4) had lower BMD than smoking and non-smoking controls (120.1 ± 44.4 mg/cm^3^ vs 148.9 ± 47.8 mg/cm3 vs 137.8 ± 39.6 mg/cm3, *P* < 0.001) the highest BMD quartile (BMD≥143.6 mg/cm^3^ in men and ≥142.1 mg/cm^3^ in women) was associated with a 36.0% decreased risk of death (HR = 0.64, 95% CI: 0.52-0.80, *P* < 0.001) compared with the lowest quartile (BMD<87.3 mg/cm^3^ in men and BMD<87.1 mg/cm^3^) in women
Romme et al (11), 2013	cross-sectional	ECLIPSE study n = 2079	respiratory disease other than COPD; significant inflammatory disease; cancer in the 5 years before study entry; evidence of alcohol, drug, or solvent abuse; previous lung surgery; chronic use of oral corticosteroids	comparison of CT-measured bone attenuation of the thoracic vertebrae between COPD patients and smoker- and nonsmoker-controls the relation of CT-measured bone attenuation to clinical parameters and inflammatory biomarkers in the COPD cohort	mean value of estimated bone attenuations of vertebra T4, T7, and T10 (in HU); ROI in the central part of vertebrae	n/a	BMD in COPD patients (164.9 ± 49.5 HU) was lover compared with smoking (183.8 ± 46.1 HU) and nonsmoking controls (212.1 ± 54.4 HU), *P* < 0.001; but after adjustment for age, sex, and pack-years of smoking the difference was not significant, *P* = 0.206 BMD correlated positively with FEV_1_ (r = 0.062, *P* = 0.014), FEV_1_/FVC (r = 0.102, *P* < 0.001), BMI (r = 0.243, *P* < 0.001), and negatively with the extent of emphysema (r = 0.090, *P* < 0.001)
Pompe et al (12), 2016	cross-sectional	NELSON trial n = 1093	self-reported moderate or poor health; recent CT scan of the thorax; current or previous cancer history; weight more than 140 kg	the association of BMD and vertebral fracture with several CT quantifications measured for lung abnormalities in patients with and without COPD	L1 vertebra and, if fractured, the closest one (in HU); ROI of only homogenous trabecular bone	yes	when Perc15 (emphysema measurement) decreased by 10 HU, BMD decreased by 1.27 HU (*P* = 0.02) BMD (*P* < 0.01) and smoking status (*P* = 0.03) were associated with the presence of a fracture COPD was not independently associated with lower BMD and fracture
van Dort et al (13), 2019	cohort study	ECLIPSE study n = 1239	respiratory disease other than COPD: chronic use of oral corticosteroids; exacerbations requiring treatment in 4 weeks prior to enrolment; significant inflammatory disease; cancer in 5 years before study entry; evidence of alcohol, drug, or solvent abuse; previous lung surgery	the association between bone attenuation and the prevalent vertebral fractures measured on CT scans with the risk of incident vertebral fractures in current and former smokers with and without COPD	mean value of estimated bone attenuations of vertebra T4 to T12 (in HU); ROI of 275 mm^3^ in all vertebrae	yes	no significant difference in the mean BMD between COPD (154.7 ± 46.8) and non-COPD subjects (157 ± 48.6), *P* = 0.0699 when only men were extracted from the study population and BMD was measured at Th12 a significant difference in BMD was found between subjects with and those without COPD (*P* = 0.0359) COPD or GOLD stage did not increase the odds for prevalent VF in multivariate models or the risk of incident VF

*Abbreviations: LAA% – low attenuation area percentage; FEV_1%_pred – forced expiratory volume in 1 second predicted; HU – Houndsfield units; VF – vertebral fracture; Perc15 – 15th percentile point; COPD – chronic obstructive pulmonary disease; BMD – bone mineral density; FVC – forced vital capacity; HR – hazard ratio; CI – confidence interval; DEXA – dual energy x-ray absorptiometry

Wilson et al also showed that the highest BMD quartile (BMD≥143.6 mg/cm^3^ in men and ≥142.1 mg/cm^3^ in women) was related to a 36.0% decreased risk of death compared with the lowest quartile (BMD<87.3 mg/cm^3^ in men and BMD<87.1 mg/cm^3^ in women; HR = 0.64, 95% CI 0.52-0.80, *P* < 0.001) (10). As for functional lung parameters, in the study by Romme et al, BMD positively correlated with FEV_1_ (r = 0.062, *P* = 0.014), FEV_1_/FVC (r = 0.102, *P* < 0.001), and body mass index (BMI) (r = 0.243, *P* < 0.001). It negatively correlated with the extent of emphysema (r = 0.090, *P* < 0.001) (11). Regarding BMD and emphysema, Pompe et al demonstrated that when Perc15 (emphysema measurement) decreased by 10 HU, BMD decreased by 1.27 HU (*P* = 0.02) (12) (Table 1).

### The association of BMD with the stage and duration of COPD, and with emphysematous type of COPD

Out of 12 studies included, seven involved only COPD patients. These studies had different aims (Supplementary Table 1(Supplementary Table 1)). Three studies measured BMD with standard CT, while four studies measured it with QCT. The majority of the studies investigated the association of BMD with the stage and duration of COPD (14-17), three studied the relationship between BMD and emphysematous type of COPD (14,17,18), one study assessed the impact of the disease dynamics on BMD (19), and one assessed the correlation between BMD measured by CT and BMD measured by DEXA, as well as determined the overall lower BMD in a selected COPD population (7). Jaramillo et al detected low BMD in 58% of all study participants, more frequently in patients with more severe COPD. Low BMD was reported in 84% of patients with the most severe COPD (17). Moreover, Hwang et al showed that the mean BMD differed significantly between the GOLD stages (GOLD 1 – 141.3 ± 55.6 HU vs GOLD 4 – 105.8 ± 45.4 HU; *P* = 0.003) (15). No association between GOLD stage and duration of COPD with BMD was demonstrated by Goto et al (14) and Wang et al (16). More precisely, there was no association between average BMD and FEV_1_. Goto et al (14) found that the progression of osteoporosis and the progression of COPD were not directly connected with each other. In the study by Wang et al, BMD wasting was 5.63 HU/per year (*P* < 0.0001) and the duration of COPD was not associated with the bone loss of the vertebral body (16).

Goto et al found that COPD, especially emphysema, was associated with both low BMD and low visceral fat, after adjusting for the history of corticosteroid use, age, the amount of overall smoking (pack-years), ongoing smoking, and disease exacerbations (14). Ohara et al showed that BMD was significantly correlated with the amount of lung parenchyma affected by emphysema (18). On the contrary, Goto et al found no association between the two (14).

Kiyokawa et al showed that the median annual changes in BMD and BMD/base were significantly greater in patients with than in those without COPD exacerbations (ΔBMD mg/mL year: -3.78 vs -0.30, *P* = 0.01) (19). In the study by Hwang et al, lower BMD, in addition to older age, decreased BMI, FEV_1_, and diffusing capacity of the lungs for carbon monoxide, was an independent predictor of all-cause mortality (HR, 1.957; 95% CI, 1.075-3.563, *P* = 0.028) (15).

### Values of BMD in COPD patients in relation to risk factors

Out of 12 studies included, from eight we were able to extract exact numerical data on BMD in COPD patients, and data on factors that could have affected the results (Table 2). QCT was used in two studies and CT was used in six. In these six studies, BMD was measured directly by positioning the region of interest (ROI) on the selected vertebra on a chest CT scan. Most studies measured BMD at the level of several arbitrarily chosen thoracic vertebrae (10,11,13-15), while two studies measured it at the level of the L1 vertebra (3,16). One study had three sites of measurement on the thoracic spine (T4, T7, and T10) (19). Four studies excluded fractured vertebrae from bone density measurements (3,10,13,15). The total number of included COPD patients in the eight studies was 7914. The patients were predominantly male (90%-100%) in six studies, while two studies had a 40% share of women (11,13). The average patients’ age ranged from 62 to 70 years. The average BMI value was available for seven studies and, in all of them, was within the normal range (18.5 to 25). The average FEV_1_% predicted in four studies was in the GOLD 2 category (50-79 FEV_1_% predicted) (10,14,15,19) and in three studies in the GOLD 3 category (30-49 FEV_1_% predicted) (11,13,16). For one study the numerical value of FEV_1_% predicted was not available, only the distribution of patients according to GOLD categories, with over 60% patients classified in the GOLD 1 category (3). Four studies reported LAA%, which ranged from 11.7 ± 12.3% to 32.6% (Q1-Q3, 24.9%-39.2%). The number of patients using oral or systemic corticosteroid therapy was reported in five studies, and was 3198 (53.06%). Only one study (14) reported two patients using osteoporosis therapy, while others did not collect this type of data. The average BMD measured with CT ranged from 100.7 ± 33.6 HU to 164.9 ± 49.5 HU; and when measured with QCT, from 120.1 ± 44.4 mg/mL to 135.8 mg/mL.

**Table 2:** Numerical values of bone density measured by both CT methods; and factors that might have contributed to the results*

Author and year	Number of COPD patients (% men)	Age (years), mean±SD	BMI mean±SD	FEV_1%_pred or GOLD stage mean±SD	Emphysematous subtype (LAA%, -950 HU) mean±SD	Patients using corticosteroids	Patients using therapy for osteoporosis	BMD in COPD patients mean±SD	Location of BMD measurement	Fractured vertebrae excluded from measurement
De Jong et al (3), 2014	437 (100)	63.2 ± 5.5	26.1 ± 3.6	GOLD 1 – 277 (63.4%) GOLD 2 – 135 (30.9%) GOLD 3 – 25 (5.7%)	N/A	N/A	N/A	100.7 ± 33.6 HU	L1 (in HU)	yes
Kiyokawa et al (19), 2012	42 (92.8) Exacerbation 13 (85%) No exacerbation 29 (96.6)	median age (Q1-Q3) 70 (65.0,76.3)	median BMI (Q1-Q3) 21 (19.5, 23.3)	median FEV1% pred. (Q1-Q3) 56.4 (41.8, 69.4)	median LAA% (Q1-Q3) Exacerbation 32.6 (24.9, 39.2) No exacerbation 31.8 (24.9, 38.1)	29	excluded	Exacerbation = 132.4 mg/ml No exacerbation = 135.8 mg/mL	T4, T7, and T10 (in mg/mL);	n/a
Wilson et al (10), 2022	4248 (100)	63.1 ± 8.6	BMI<18.5 – 106 (2.5%) 18.5≤BMI<25 – 1369 (32.22%) 25≤BMI<30 – 1435 (33.78%) BMI≥30 – 1338 (31.5%)	57.6 ± 22.7	11.7 ± 12.3	1880 (44.26%)	N/A	120.1 ± 44.4 mg/mL	x̄ of T6-L1 (in mg/cm^3^)	yes
Romme et al (11), 2013	1634 (64)	63.3 ± 7.1	26.5 ± 5.8	48.5 ± 15.9	17.7 ± 12.3	1186 (73%)	N/A	164.9 ± 49.5 HU	x̄ of T4, T7, and T10 (in HU)	n/a
van Dort et al (13), 2019	999 (61.9)	62.8 ± 7.0	25.6 ± 4.6	49.6 ± 15.7	N/A	Excluded	N/A	151.3 ± 46.7 HU	x̄ of T4 to T12 (in HU)	yes
Goto et al (14), 2018	103 (93.2)	69.8 ± 7.8	22.7 ± 2.0	66.8. ±20.2	24.6 ± 13.9	8 (7.8%)	2 (1.9%)	Th_4,7,10_ – 132.2 ± 43.1 HU Th_1-12_ – 138.0 ± 43.0 HU	x̄ of T4, T7, and T10 (in HU)	n/a
Hwang et al (15), 2020	322 (92.55)	65.6 ± 7.7	22.9 ± 4.3	61.9 ± 20.3	N/A	95 (29.5%)	N/A	128 ± 50 HU	x̄ of three consecutive thoracic vertebral bodies at the level of the main coronary artery (in HU)	yes
Wang et al (16), 2022	129 (100)	69.7 ± 10.0	N/A	median FEV1% pred. (Q1-Q3) 40.3 (24.5, 60.2)	N/A	N/A	N/A	113.8 ± 43.9 HU	L1 (in HU)	n/a

*Abbreviations: LAA% – low attenuation area percentage; FEV_1%_pred – forced expiratory volume in 1 second predicted; HU – Houndsfield units; x̄ – average.

## DISCUSSION

To the best of our knowledge, this review was the first to systematically evaluate studies that examined the effect of COPD on BMD assessed by chest CT. COPD patients regularly undergo a chest CT scan for a more detailed analysis and categorization of the disease. CT is also performed in these patients as a part of the national program for the early detection of lung cancer due to shared risk factors. Therefore, CT may serve as a surrogate method for BMD measurement instead of DXA, since it may provide more valuable information within a single examination, thus reducing the radiation exposure.

CT and QCT have similarities and differences. Although standard CT cannot directly calculate BMD, it can provide valuable and precise information regarding the structure of the measured bone (x-ray attenuation in HU), which may indicate bone loss or other skeletal changes (20). It has a higher radiation dose than QCT, but when it is performed in the diagnostics of COPD, it is a quick and easy method to opportunistically examine bone density. QCT was specially designed to measure BMD and has to be done as a separate examination. It measures BMD in three dimensions and is particularly useful for evaluating the trabecular bone (21). Moreover, it has better sensitivity than DXA and better correlates with clinical outcomes related to fractures than conventional CT or DXA (22). Despite this, DXA is still the standard method for assessing BMD due to lower radiation exposure and wider availability (23).

The exact mechanisms that link COPD and osteoporosis are unclear. Osteoporosis is either a systemic manifestation of COPD with a causal link, or comorbidity due to shared risk factors (eg, older age and smoking). A number of pathophysiologic processes have been proposed to link osteoporosis with COPD, including vitamin D insufficiency or deficiency, disruption of the osteoprotegerin/receptor activator of NF-kB (RANK)/RANK ligand pathway, and systemic inflammation (11).

Among several factors that impact bone turnover, and thus BMD, patient’s age is considered the main determinant (4,6). However, in the studies included in this review, although the average age ranged from 60 to 70 years, the average BMD highly varied from study to study. For example, in six studies, where it was measured with CT, BMD ranged from 100 HU to 164 HU, and in two studies, where it was measured with QCT, it was 120 mg/mL and 135 mg/mL, respectively. Several studies have shown that the average BMD gradually decreases craniocaudally; therefore, the location of the BMD measurement might have affected the result (6,24). For example, six studies measured the average BMD of several arbitrarily chosen thoracic vertebrae, while only two studies measured it at the level of the L1 vertebra. These two studies obtained the lowest average BMD values (100.7 ± 33.6 HU and 113.8 ± 43.9 HU). Also, some studies involved the measurement of the T4 vertebra, whose expected BMD is higher than that of the middle or lower group of thoracic vertebrae. Only four studies excluded fractured vertebrae from the measurement, while this information was not provided in the other studies. The BMD of fractured vertebrae is higher, so this could have affected the average BMD values of COPD patients (25). Standard locations of BMD measurements with DXA include vertebral bodies L1 to L4, femoral neck, or total hip measurement (23). Less often they include the forearm or total body (23). The majority of the included studies measured BMD at the thoracic vertebrae in addition to the L1 vertebra, but some measured it only at the thoracic vertebrae. The thoracic spine can be divided into three regions: upper (T1-T4), middle (T5-T8), and caudal region (T9-T12). The BMD of the caudal region is closest to the BMD of the L1 vertebra measured by DXA, not only anatomically but also biomechanically (26). Therefore, measuring BMD at the specified level can be a good indicator of systemic bone health and fracture risk (27). The BMD of the thoracic spine must be interpreted with caution because it is often affected by spondylodegenerative changes, fractures, or scoliosis (28). Consequently, current DXA studies suggest that BMD measurements can provide additional information for assessing osteoporosis, but they cannot serve for diagnostic purposes (29).

Although the main determinant of BMD is age, sex, and BMI are shown to have an impact (4). The majority of patients in our review were men (64% to 100%), so we were unable to observe the influence of sex on BMD. Similarly, most of the included patients were in the normal BMI range or slightly overweight, which prevented us from determining the impact of BMI on the BMD value.

Some studies showed the impact of COPD severity, expressed in GOLD stage, on average BMD (11,15,17,30). Based on the values of FEV_1_% predicted, the patients included in our study were either GOLD 2 or GOLD 3 category. Although we did not observe that GOLD 3 patients had lower BMD than GOLD 2 patients, it has to be taken into account that the included studies differed in terms of BMD measurement locations and other parameters that impact BMD. Romme at al also concluded that sex, race, or the method of emphysema and BMD quantification might have contributed to the lower correlation coefficients between FEV1, FEV1/FVC, and BMD observed in their study in comparison with other studies (11).

The use of corticosteroid therapy has been proven to affect the BMD value, more precisely the development of osteoporosis (31). A review paper (5) revealed that although several studies showed the influence of systemic corticosteroid therapy on BMD in COPD patients, these studies included a small number of COPD patients on systemic corticosteroid therapy. In COPD patients, however, the possible influence of inhaled corticosteroids (ICS) on BMD is more important. Some studies indicated that ICS was associated with a modest but significant fracture risk, and was dose-dependent (32). In the studies included in our review, most of the patients did not take corticosteroid therapy or this information was not specified. Although in the study by Romme et al the majority of patients did take corticosteroid therapy (73%), the study obtained the highest average BMD values (164.9 ± 49.5 HU), possibly due to the large number of patients, the inclusion of the T4 vertebra in the measurement, and the lack of information on the cumulative dose and the duration of therapy (11). Although several studies have suggested a connection between the emphysematous type of COPD and reduced bone density, the value of LAA% was available in only four studies and it ranged from 11.7 ± 12.3 to 32.6 (24.9-39.2). The studies did not note a difference in BMD in patients with higher LAA% values, probably due to heterogeneous other parameters that affect BMD, as was the case with sex and BMI. Finally, one of the important factors that affect BMD is anti-osteoporotic therapy (5,14,19). Unfortunately, only one study reported the number of patients using therapy for osteoporosis (14). To summarize, the average BMD value of the patients in the studies from which we were able to extract this type of data entered the category of osteopenia or even osteoporosis (33,34), which is in accordance with their average age, regardless of the COPD status.

There are several limitations to our study, the most important one being the relatively small number of total studies included. Six studies were cross-sectional, while other six were cohort studies with a follow-up period of no longer than 10 years. Furthermore, the included studies, although containing important data, were heterogeneous mostly regarding the study population, so we were unable to extract all relevant data for COPD patients to perform statistical analysis.

In conclusion, although in several studies COPD was associated with a decrease in BMD, other studies found that COPD, especially in its milder forms, was not strongly associated with osteopenia or osteoporosis of the thoracic and lumbar spine. Osteoporosis leads to bone fractures, and thoracic vertebral fractures can worsen already damaged lung functions in COPD patients. Estimation of bone trabecular density of the thoracic spine on standard chest CT scans requested for COPD evaluation allows deeper insight into bone health and potential estimation of fracture risk. This review presents current evidence on the relationship between BMD and COPD, but it also reveals gaps in the knowledge on the possible effect of disease severity and corticosteroid therapy on BMD in COPD patients.
