# Supplementary material for: What can we learn about bone density in COPD patients from a chest CT? A systematic review
Source: Croat Med J. 2024 Oct;65(5):440–9. doi: 10.3325/cmj.2024.65.440 (PMC11568385; doi:10.3325/cmj.2024.65.440)
Supplement: Supplementary Table 1 [file CroatMedJ_65_s011.pdf]

Supplementary Table 1. Articles with only COPD patients – investigation and findings*

Author and year	Study design	Participants	Exclusion criteria	Research question	Location of BMD measurement and method	Fractured vertebra excluded	Outcomes regarding BMD
Kiyokawa et al (19), 2012	cohort	Patients with COPD at Kyoto University Hospital, n = 42	history of respiratory disease other than COPD<br>malignancy within the previous 5 years<br>history of bone disease<br>currently receiving osteoporosis treatment<br>currently receiving oral corticosteroids<br>receiving rehabilitation during the study<br>home-oxygen therapy<br>a new shadow or vertebral fracture in the follow up period	impact of exacerbations of COPD on BMD, lung function, and empysematous change in a follow-up period of 2 years on chest CT scans	T4, T7, and T10 (in mg/mL); ROI was placed at the largest possible area in the anterior portion of the vertebral body	n/a	the median annual changes in BMD and BMD/base were significantly greater in patients with than in those without exacerbations<br>ΔBMD mg/mL year: -3.78 vs -0.30, $P = 0.01$<br>ΔBMD/base %: - 5.41 vs -0.60, $P = 0.02$

Romme et al (7), 2012	cross-sectional	Patients with moderate to very severe COPD in Catharina Hospital (Eindhoven, The Netherlands), n = 58	malignancy in the last 5 years hypo- or hyperthyroidism; hypogonadism; inflammatory bowel disease; autoimmune diseases alcohol abuse nonvertebral fracture in the last 12 months pulmonary diseases other than COPD such as pulmonary fibrosis or bronchiectasis use of oral corticosteroids or antibiotics 4 weeks before study enrollment use of anti-osteoporotic medication osteopenia	to measure the average attenuation of T4, T7, and T10 on chest CT and correlate these measurements with DXA in patients with COPD	attenuation of T4, T7, and T10 (in mg/cm^3); ROI in the central part of the vertebrae	yes	high correlation between CT-measured bone attenuation and lower BMD assessed on DXA ($r = 0.827$, $P < 0.001$)
Goto et al (14), 2018	cohort	Hokkaido COPD cohort study, n = 103	bronchial asthma, bronchiectasis,	to determine whether the progression of osteoporosis is	mean value of estimated bone attenuations	n/a	average BMD was not associated with FEV_1 average BMD was not associated with LAV%.

			or bullous lung disease<br>active tuberculosis<br>history of lung cancer, cystic fibrosis, allergic alveolitis, or pulmonary fibrosis<br>history of lung resection<br>long-term oxygen therapy for 12 h or more per day<br>exacerbation experienced within a month before enrollment<br>withdrawal of consent	synchronized with that of COPD.<br>the relationship of annual change in BMD with annual changes in pulmonary function and emphysematous lesions	of vertebra T4, T7, and T10 (in HU); measured using custom software		progression of osteoporosis and that of COPD are not directly related or synchronized with each other
Hwang et al (15), 2020	cohort	COPD patients from Korean Obstructive Lung Disease (KOLD) cohort, n = 322	co-existing significant illnesses (eg, malignancy, congestive heart failure, chronic renal failure, diabetes with severe	to assess the association between thoracic vertebral bone density measured on chest CT and clinical	mean BMD was calculated from three consecutive thoracic vertebral bodies at the level of the	yes	BMD on baseline chest CT was 128 ± 50 HU<br>BMD differed significantly between different GOLD stages (*P* = 0.003); GOLD 1 – 141.3 ± 55.6 vs GOLD 4 – 105.8 ± 45.4<br>in a multivariate Cox analysis, lower BMD (HR, 1.957; 95%

			complications, significant inflammatory diseases such as rheumatoid arthritis)<br>recent exacerbation<br>other respiratory illness	variables and patient mortality	main coronary artery (in HU); ROI as large as possible in the anterior portion of the vertebrae		CI, 1.075-3.563, $P = 0.028$) along with older age, lower BMI, lower FEV_1, and lower DLCO, was an independent predictor of all-cause mortality
Wang et al (16), 2022	retrospective cohort	COPD patients with two diagnostic chest CTs with an interval of at least 3 months, n = 129	chronic liver disease, end-stage renal disease and active cancer<br>bronchial asthma, interstitial pneumonia, bronchiectasis, thoracic or upper lumbar vertebrae degenerative disease<br>history of vertebral operation	to investigate BMD change over time	L1 vertebra on chest CT (in HU); ROI at the mid-vertebral body	n/a	bone density loss was 5.63 HU/per year ($P < 0.0001$)<br>the duration of COPD was not associated with vertebral bone loss
Jaramillo et al (17), 2015	cross-sectional	Patients from COPDGene, n = 3321	N/A	to investigate the relationship of age, sex, race, steroid use, smoking,	mean BMD from a minimum of three vertebral	yes	low vBMD was present in 58% of all participants, more frequently in those with worse COPD; 84% among participants with very severe COPD.

				and COPD to reduced bone density and vertebral fractures	bodies from T6 to L1 (expressed in mg/cm^3); automated ROI of the trabecular vertebral bone		vertebral fractures were present in 37% of all participants and were associated with lower vBMD at each GOLD stage of severity<br>COPD, especially emphysema, was associated with both low vBMD and VF after adjustment for steroid use, age, pack-years of smoking, current smoking, and exacerbations.<br>calibrated QCT identified more participants with abnormal values than standard DEXA in a subset of participants, and correlated well with prevalent fractures.
Ohara et al (18), 2008	cross-sectional	Male patients with stable COPD, n = 65	history of other respiratory disease<br>history of chest surgery<br>cancer in the 5 years before study entry<br>history of bone disease<br>current or previous oral or inhaled CS therapy	to measure both CT scan density in the vertebral bones and LAA% in the lungs using chest CT images.<br>to investigate the relationship between emphysema and osteoporosis using	T4, T7, T10, and L1 (in mg/mL); ROI as large as possible in the anterior portion of vertebrae	n/a	the extent of pulmonary emphysema significantly correlated with reduced bone density.

			medicines that influence bone metabolism	quantitative CT scan parameters			

*Abbreviations: LAA% – low attenuation area percentage; $FEV_{1\%}$pred – forced expiratory volume in 1 second predicted; HU – Houndsfield units; VF – vertebral fracture; Perc15 – 15th percentile point; COPD – chronic obstructive pulmonary disease; BMD – bone mineral density; vBMD – volume bone mineral density; FVC – forced vital capacity; HR – hazard ratio; CI – confidence interval; DEXA – dual energy x-ray absorptiometry
